# Analysis of four toxic metals in a single rice seed by matrix solid phase dispersion -inductively coupled plasma mass spectrometry

**DOI:** 10.1038/srep38472

**Published:** 2016-12-06

**Authors:** Xiufen He, Lixia Chen, Xin Chen, Huamei Yu, Lixu Peng, Bingjun Han

**Affiliations:** 1 Analysis & Testing Center, Chinese Academy of Tropical Agricultural Sciences, Hainan Provincial Key Laboratory of Quality and Safety for Tropical Fruits and Vegetables, Haikou, Hainan 571101, China; 2 Mathematical Department, Qiongtai Teachers College, Haikou, Hainan 571100, China; 3 Environment and Plant Protection Institute, Chinese Academy of Tropical Agricultural Sciences, Haikou, Hainan 571101, China; 4 College of Environment and Plant Protection, Hainan University, Haikou, Hainan 570228, China

## Abstract

Toxic metals in rice pose great risks to human health. Metal bioaccumulation in rice grains is a criterion of breeding. Rice breeding requires a sensitive method to determine metal content in single rice grains to assist the variety selection. In the present study, four toxic metals of arsenic (As), cadmium (Cd), chromium (Cr) and lead (Pb) in a single rice grain were determined by a simple and rapid method. The developed method is based on matrix solid phase dispersion using multi-wall carbon nanotubes (MWCNTs) as dispersing agent and analyzed by inductively coupled plasma mass spectrometry. The experimental parameters were systematically investigated. The limits of detection (LOD) were 5.0, 0.6, 10 and 2.1 ng g^−1^ for As, Cd, Cr, and Pb, respectively, with relative standard deviations (n = 6) of <7.7%, demonstrating the good sensitivity and precision of the method. The results of 30 real world rice samples analyzed by this method agreed well with those obtained by the standard microwave digestion. The amount of sample required was reduced approximately 100 fold in comparison with the microwave digestion. The method has a high application potential for other sample matrices and elements with high sensitivity and sample throughput.

Rice is the most important food for the world population^1^. However, the toxic metallic elements in rice grains transferred from the environment and agricultural input^2^ often pose great risks to food safety and human health because of their toxicity, persistence, and non-degradability^3^. Those toxic metals include arsenic (As), cadmium (Cd), chromium Cr), and lead (Pb). In recent years, many researches focused on breeding rice varieties that enriched less metal elements from the environment. Therefore, the development of a rapid and simple method to determine the metal elements in rice seed samples is particularly important for the rice breeding. Moreover, the rice seeds are very valuable for breeding, especially for the space breeding and gene-modified breeding. Accurate determination of trace metal elements in single rice grains is particularly important. Such sensitive methods are necessary tools to aid rice breeding to select low metal bioaccumulation varieties. Generally, the metal elements need to be digested and extracted from the complex rice sample matrix via various extraction methods^4^,^5^,^6^,^7^. In fact, numerous methods including wet-digestion, dry-ashing^8^, microwave assisted extraction^9^,^10^, and cloud point extraction^11^,^12^ have been used for the extraction and preconcentration of solid samples. However, these extraction methods are usually tedious and time-consuming, consume high amounts of energy, and are unsuitable for small amount rice seed samples.

Matrix solid-phase dispersion (MSPD) is a sample-preparation technique to disperse samples using a selective solid-phase for the extraction of analytes from solid, semisolid, and viscous matrices^13^,^14^. With the advantages of exhaustive extraction and single-step clean-up of analytes, MSPD has been widely applied for the preparation of various samples, including food^15^,^16^, plant^17^, medicine^18^, biological^19^ and environmental^20^ samples. In fact, MSPD is mainly applied for the extraction of organic compounds, including pesticides^21^, auxins^22^, endocrine disrupting chemicals^23^, polycyclic aromatic hydrocarbons^24^ and persistent organic pollutants^25^. However, the common selective MSPD sorbents such as multi-walled carbon nanotubes^18^, alumina^16^, C18^26^, silica^27^, and florisil^28^ contain metal impurities which limit the application of MSPD in the extraction of metal elements. For example, multi-wall carbon nanotubes (MWCNTs) may contain up to 30 wt% of residual metal impurities (nickel, iron and etc.)^29^. Therefore, arsenic and mercury^30^ were most suitable for elemental extraction with MSPD, but other metal elements were quite limited. Fortunately, the residual metal and impurities in MWCNTs can be purified by methods such as acid treatment^31^, microwave irradiation^32^, photochemical vapor generation^29^ and plasma-thermal purification^33^. The lower matrix effect of purified MWCNTs enhanced the extraction and concentration of the target metal elements via MSPD.

The aim of this work was to investigate the potential of MSPD for the extraction of multiple metal elements in a single rice seed. Acid treatment was chosen for the purification of commercial MWCNTs, and washed MWCNTs were sequentially applied for the MSPD extraction of toxic metal elements (As, Cd, Cr and Pb) in a single rice seed. The proposed sample preparation method coupled with ICP-MS was validated and successfully applied to analysis of metal elements in rice samples.

## Results and Discussion

### Characterization of pretreated MWCNTs

**Figure 1:**
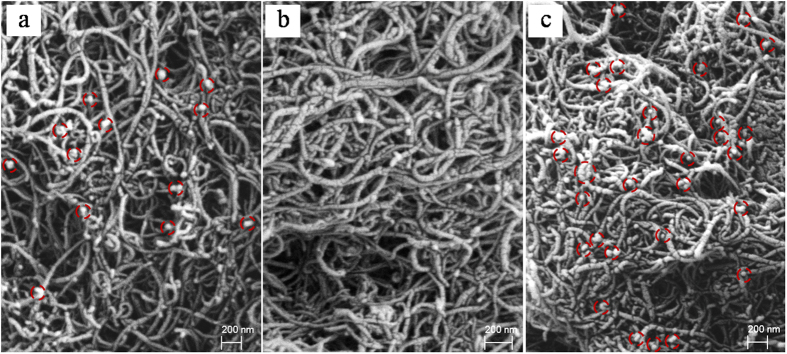
SEM images of MWCNTs: (**a**) prior to purification, (**b**) after purification (**b**), and (**c**) in a mixture with rice sample.

Treated and untreated MWCNTs samples were first characterized by SEM (Fig. 1). There were some nano-scale metal particles presented in the untreated SEM image and these metal particles were encapsulated in the CNT central canal (Fig. 1a). However, these nano-scale metal particles were hardly found after purification, and were thought to be removed in the cleanup process (Fig. 1b). The purified MWCNTs did not show any damage and morphological changes such as length shortening, which meant that the pretreatment served as purification, but did not change or modify the W-MWCNTs characteristics. When the W-MWCNTs were ground with the rice sample, as demonstrated in Fig. 1c, the MWCNTs dispersed the rice sample completely. Most importantly, the MWCNTs generated numerous carbon nanofibers, which prevented the aggregation of the mixture. Therefore, MWCNTs were used as a good solid matrix to disperse the rice samples.

**Figure 2:**
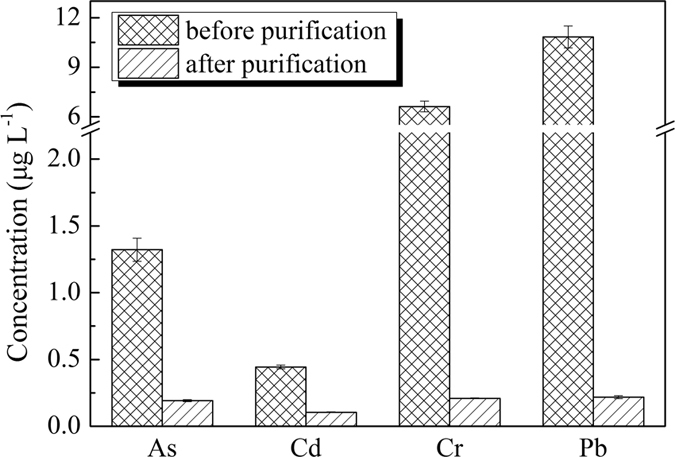
Comparison of concentrations of the four metals in the MWCNTs before and after the acid purification treatment.

In order to further investigate the removal of the metal elements from the W-MWCNTs, the four heavy metal element impurities in MWCNTs were quantitatively determined before and after purification. Treated and untreated MWCNTs (0.02 g) were digested by microwave digestion (power: 800 W; temperature: 160 °C; time: 20 min) with the concentrated nitric acid (5 mL) and hydrogen peroxide solution (1 mL). The purification treatment showed that approximately 85%, 76%, 96% and 97% of As, Cd, Cr and Pb were removed from MWCNTs, respectively, as determined by ICP-MS (Fig. 2.). The purified MWNCTs were ready for uses in the remainder of MSPD experiments.

### Optimization of MSPD for metal elements

For optimization of the MSPD experimental conditions used for extraction, the standard rice sample GBW10010 was used for sequential assessment of the heavy metals. The extraction efficiency was evaluated from a comparison of the obtained and certified values of heavy metals in GBW10010.

**Figure 3:**
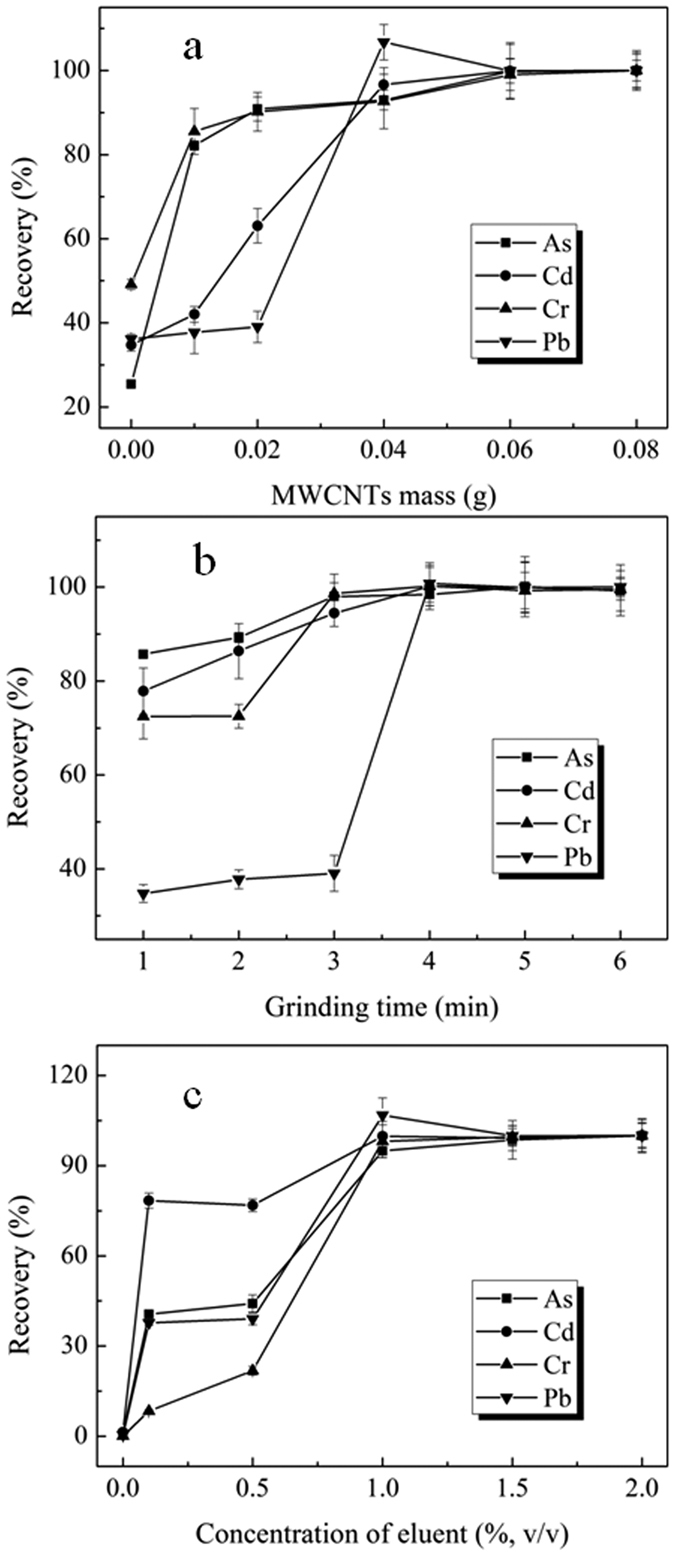
Optimization of the MSPD: (**a**) Effects of W-MWCNTs mass on the recoveries of the 4 metal elements; (**b**) Effects of grinding time on the recoveries of the 4 metal elements; (**c**) Effects of concentration of eluent on the recoveries of the 4 metal elements.

MWCNTs were used as solid supports for heavy metal extraction in the rice samples, where the mass of the MWCNTs strongly affected the extraction efficiency of the analytes. In this work, the effect of the amount of MWCNTs on the extraction efficiency was first investigated. Figure 3a shows that in comparison with the no MWCNTs controls, the extraction efficiencies of all four target metals in the 0.02 g rice sample increased with the use of MWCNTs. Average recoveries of each analyte reached to a plateau in the range of 0.04–0.08 g of MWCNTs. A lower amount of the MWCNTs did not produce efficient dispersion of the analytes. Therefore, a MWCNTs mass of 0.04 g was chosen for all subsequent extraction in this work.

The grinding time of MSPD for the analytes is another critical parameter. All recoveries of the analytes increased as the grinding time increased from 1 min to 4 min, followed by a plateau (Fig. 3b). The grinding time of 4 min was then applied in the subsequent experiments. It is noteworthy that in a preliminary experiment, two national standard rice samples were digested at 160 °C and 180 °C. The average recoveries of As at 160 °C were approximately 100%, but were only approximately 70% at 180 °C (data not shown), which indicated that the high digestion temperature resulted in the loss of As element. Therefore, the samples were ground at ambient temperature in the MSPD method rather than high temperature acid digestion in the traditional method. This is another important advantage of the MSPD method.

It is well known that aqua regia (HCl:HNO_3_, 3:1) has been used as an eluent for the extraction of heavy metals. Therefore, aqua regia was chosen as the eluent and the effects of aqua regia at various concentrations were also studied, as shown in Fig. 3c. The extraction efficiencies of the analytes were below 1% without using acid, and increased to 95% with the use of 1% or greater amounts of acid. Therefore, an eluent containing 1% (v/v) aqua regia was used for the remainder experiments.

### Analytical performance

The proposed method was evaluated under the optimal conditions (Table 1). The linear correlation coefficients of determination for calibration curves were better than 0.999 for all four metal elements at a trace concentration. The limit of detection (LOD), defined as the analyte concentration equivalent to three standard deviations of 11 measurements of a blank solution (HNO_3_, 2% v/v), was 5.0, 0.6, 10 and 2.1 ng g^−1^ for As, Cd, Cr and Pb, respectively. The precision of the proposed method was assessed by performing replicate analyses of the certified reference material (CRM) samples. The precision of the method was expressed as relative standard deviations (RSDs, n = 6), and all was less than 7.7% for the tested metal elements.

### Interference

The dispersion and extraction efficiency of the proposed method was limited by the capacity of the MWCNTs material. Interference may occur due to competition from other heavy metal elements. To evaluate the interference, the effects of several potential interfering metal elements were investigated. The standard rice sample (GBW10010) was used with the addition of a high concentration of interfering metal elements (Mg, Zn, Cu, Fe, Mn, Mo, Ce, Ni, and Se). The results showed no obvious interference observed from these target metal ions even at concentrations as high as 10 mg g^−1^ (Table 2).

### Sample analysis

**Figure 4:**
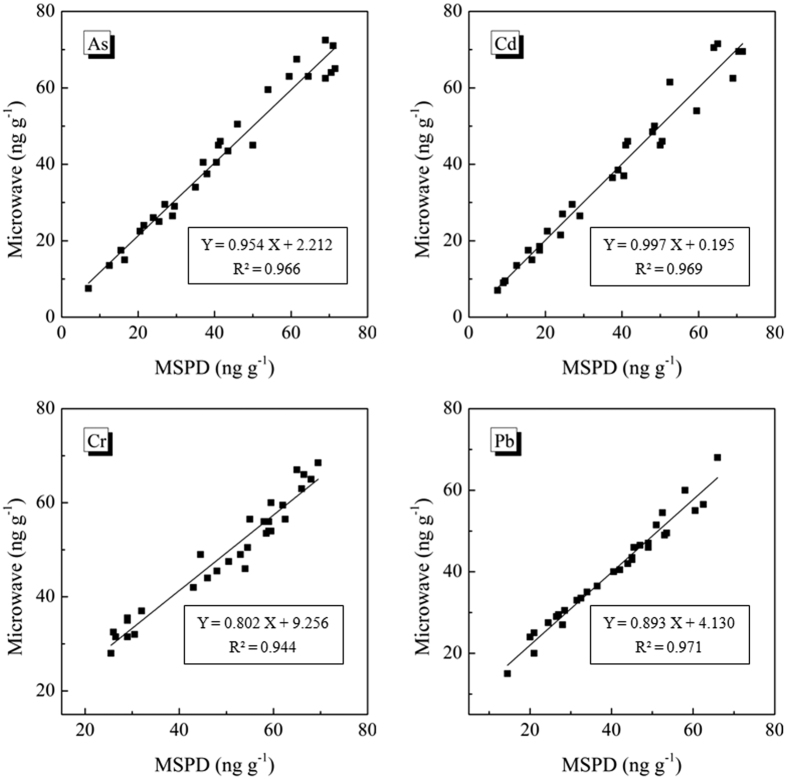
Comparison between the MSPD method and the microwave digestion method for the analysis of As, Cd, Cr and Pb in single rice grains.

Since there were no rice seed samples with certified value of the metal elements, the accuracy of the proposed method was first evaluated by analysis of four CRMs (GBW10010, GBW10043, GBW10044, and GBW10045). The t-test showed that all the analytical results produced by the proposed method were not significantly different from the certified value at a 95% confidence level (Table 3). Further, the applicability of the method was compared with the standard microwave digestion method for metal elements. The 30 real world rice seed samples were analyzed by the developed MSPD extraction method (sample amount: about 0.02 g of a single seed) and microwave digestion (sample amount: 2 g). The results were not significantly different between the two methods (Fig. 4). However, the amount of sample required was reduced about 100 fold in comparison with the standard microwave digestion.

## Conclusions

A simple and rapid solid sampling platform based on purified MWCNTs and assisted MSPD was developed for the simultaneous determination of trace amounts of As, Cd, Cr and Pb in a single rice seed sample. Compared with other methods for elemental metal analysis in rice samples, this method demonstrated several advantages such as allowing single seed analysis, use of less chemicals, lower energy consumption, simplicity and low cost. In future works, this method can be used to analyze other sample matrices and elements with high sensitivity and throughput.

## Methods

### Reagents

All chemicals were of analytical grade purity or greater. High purity 18.2 MΩ·cm ultrapure water was produced by a Milli-Q water purification system (Millipore, USA). High purity HNO_3_, HCl, and other reagents were obtained from Guangzhou Chemical Reagent Factory (Guangzhou, China). Standard stock solutions (1000 mg L^−1^) of the four elements were purchased from the National Research Center of China (NRCC, Beijing, China). Working standard solutions were obtained by stepwise dilution of the standard stock solutions and stabilized in 0.5% (v/v) HNO_3_. Multi-wall carbon nanotubes (Purity >95 wt%; 10–20 o.d. × 10–30 μm length) were obtained from Chengdu Organic Chemicals Co. Ltd (Chengdu, China). Four certified reference material (CRM) rice samples (GBW10010, GBW10043, GBW10044, and GBW10045) were purchased from the National Standard Center of China (Beijing, China) to validate the accuracy of the proposed method. Rice seed samples were purchased from a local market and were stored at 4 °C until analysis.

### Pretreatment of MWCNTs

MWCNTs samples contain many residual metal impurities. Therefore, commercial MWCNTs were washed with HNO_3_ and H_2_SO_4_ to remove the metal impurities in the initial experiment as described in previous methods^31^. Briefly, commercial MWCNTs (2 g) were added to a reaction chamber together with acid solvent (300 mL, HNO_3_:H_2_SO_4_, 1:1), and then the reaction vessels were subjected to sonication for 6 h. After the reaction, the mixture was centrifuged at 15000 rpm for 5 min. After carefully removing the aqueous phase using a syringe, the solid MWCNTs phase was diluted and rinsed with ultrapure water until neutral, and then dried at 60 °C for future use. Scanning electron microscopy (SEM) was used to characterize the washed MWCNTs (W-MWCNTs) before and after the treatment. In order to determine the removal efficiencies of metal elements from MWCNTs, ICP-MS was used to quantify the metal elements before and after the acid treatment.

### Sample preparation

**Figure 5:**
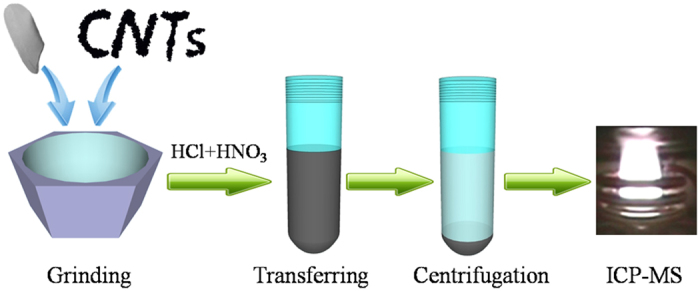
Schematic of the analytical procedures.

The MSPD procedure was performed for a single rice seed sample according to the schematic of the sample preparation illustrated in Fig. 5. A single rice seed (about 0.02 g) and W-MWCNTs (0.02 g) were weighed into an agate mortar and blended for around 5 min using an agate pestle to obtain a homogeneous mixture. This mixture was then quantitatively transferred into a 10 mL PTFE centrifuge tube, to which 1% HNO_3_:HCl (3:1, v/v, 2 mL) was added. After blending, the mixture was centrifuged at 15000 rpm for 2 min. Finally, the liquid phase was collected and subjected to ICP-MS analysis directly.

### ICP-MS conditions

The NexION 300X ICP-MS (PerkinElmer, Inc., Shelton, CT) was used throughout this work. The optimized parameters and details of the instrumental settings were listed in Table 4. The water stream and metal sample solution were introduced into the system by a peristaltic pump at the flow rate of 0.5 mL min^−1^. Rhodium (Rh, 25 μg L^−1^) was added as an internal standard to minimize the instrumental signal fluctuation and matrix effects during the measurement of the samples.

## Additional Information

**How to cite this article**: He, X. *et al*. Analysis of four toxic metals in a single rice seed by matrix solid phase dispersion -inductively coupled plasma mass spectrometry. *Sci. Rep.* **6**, 38472; doi: 10.1038/srep38472 (2016).

**Publisher's note:** Springer Nature remains neutral with regard to jurisdictional claims in published maps and institutional affiliations.

## Figures and Tables

**Table 1:** Analytical characteristics of the proposed method.

Analyte	Calibration equation	R^2^	RSD (%, n = 6)	LOD (ng g^−1^)
As	I = 234 C + 46^a^	0.999	7.7	5.0
Cd	I = 692 C + 55	0.999	5.5	0.6
Cr	I = 2454 C + 1842	0.999	6.4	10
Pb	I = 9435 C + 516	0.999	5.4	2.1

^a^I stands for intensity, C stands for concentration.

**Table 2:** Effect of coexisting metallic ions on recoveries of the four target metals.

Coexisting ions	Spiked (mg g^−1^)	Average recovery (%)			
		As	Cd	Cr	Pb
Mg	10	95	108	93	107
Zn	10	94	95	100	115
Cu	10	100	94	95	114
Fe	10	101	99	115	105
Mn	10	107	101	109	101
Mo	10	112	101	115	101
Ce	10	105	111	93	98
Ni	10	111	105	115	108
Se	10	92	102	111	103

**Table 3:** Comparison of the results measured by the MSPD method with the certified values.

Sample		Certified (μg g^−1^)	Found (μg g^−1^)^a^
GBW10010	As	0.102 ± 0.008	0.106 ± 0.007
	Cd	0.087 ± 0.005	0.083 ± 0.019
	Cr	(0.090)^b^	0.098 ± 0.012
	Pb	0.080 ± 0.030	0.074 ± 0.011
GBW10043	As	0.114 ± 0.018	0.115 ± 0.016
	Cd	0.012 ± 0.003	0.012 ± 0.002
	Cr	0.140 ± 0.05	0.130 ± 0.015
	Pb	0.075 ± 0.025	0.081 ± 0.005
GBW10044	As	0.120 ± 0.03	0.128 ± 0.023
	Cd	0.018 ± 0.002	0.018 ± 0.002
	Cr	0.170 ± 0.05	0.155 ± 0.021
	Pb	0.090 ± 0.030	0.093 ± 0.004
GBW10045	As	0.110 ± 0.020	0.098 ± 0.010
	Cd	0.190 ± 0.020	0.201 ± 0.016
	Cr	(0.140)	0.151 ± 0.015
	Pb	0.070 ± 0.023	0.069 ± 0.002

^a^Average ± standard deviation of three trials.

^b^The numerical value in the bracket is for reference.

**Table 4:** Operating conditions for the argon ICP-MS.

**ICP system**	RF power (W)	1300
	sampler (orifice diameter, mm)	nickel, 1.1
	skimmer (orifice diameter, mm)	nickel, 0.9
	plasma gas flow rate (L min^−1^)	16
	auxiliary gas flow rate (L min^−1^)	1.2
	carrier gas flow rate (L min^−1^)	0.95
	injector nozzle (tip i.d., mm)	2.0
**MS system**	points/mass	1
	resolution (amu)	0.7
	sweeps/reading	20
	readings/replicates	1
	replicates	3
	dwell time/mass (ms)	50
	integration time (ms)	1000
